# Deciphering the role of a SINE-VNTR-*Alu* retrotransposon polymorphism as a biomarker of Parkinson’s disease progression

**DOI:** 10.1038/s41598-024-61753-5

**Published:** 2024-05-13

**Authors:** Alexander Fröhlich, Abigail L. Pfaff, Ben Middlehurst, Lauren S. Hughes, Vivien J. Bubb, John P. Quinn, Sulev Koks

**Affiliations:** 1 Department of Pharmacology and Therapeutics, Institute of Systems, Molecular and Integrative Biology, University of Liverpool, Liverpool, UK; 2 Perron Institute for Neurological and Translational Science, Perth, WA Australia; 3 Centre for Molecular Medicine and Innovative Therapeutics, Murdoch University, Perth, WA Australia

**Keywords:** Neurology, Pathogenesis, Parkinson's disease, Gene expression, Gene regulation, Genotype

## Abstract

SINE-VNTR-*Alu* (SVA) retrotransposons are transposable elements which represent a source of genetic variation. We previously demonstrated that the presence/absence of a human-specific SVA, termed SVA_67, correlated with the progression of Parkinson’s disease (PD). In the present study, we demonstrate that SVA_67 acts as expression quantitative trait loci, thereby exhibiting a strong regulatory effect across the genome using whole genome and transcriptomic data from the Parkinson’s progression markers initiative cohort. We further show that SVA_67 is polymorphic for its variable number tandem repeat domain which correlates with both regulatory properties in a luciferase reporter gene assay in vitro and differential expression of multiple genes in vivo. Additionally, this variation’s utility as a biomarker is reflected in a correlation with a number of PD progression markers. These experiments highlight the plethora of transcriptomic and phenotypic changes associated with SVA_67 polymorphism which should be considered when investigating the missing heritability of neurodegenerative diseases.

## Introduction

Transposable elements (TEs) constitute about 45% of the human genome as a consequence of many ancient insertion events, nevertheless many elements are no longer active for mobilisation^1,2^. However, the term “jumping genes” starting with the work by Barbara McClintock in the 1950s is of tremendous interest as some TE classes remain active today which contribute not only to genome variability, function and evolution, but can also cause or affect progression of disease^3–7^. Several families of retrotransposons belong to these active classes, including members of the long interspersed nuclear elements (LINEs), *Alu*, and SINE-VNTR-*Alu* (SVA), which propagate via a “copy and paste” mechanism called target-primed reverse transcription^2,8^. Full-length SVA retrotransposons, approximately 0.7–4 kb in length, are hominid-specific elements and contain five key domains including a 5’ CT hexamer repeat (CCCTCT), an inverted *Alu*-like domain, a GC-rich variable number tandem repeat (VNTR) region, a SINE-R domain and a canonical poly-A signal (AATAAA) at the 3’ end which is capped with a poly-A tail^1,9^. They make up 0.13% of the total human genome with 2700–3000 elements present in the reference human genome which are split into subclasses A-F1 based on evolutionary age and the composition of the SINE-R domain^10^.

It has been demonstrated that SVAs harbour transcription factor (TF) binding sites and thus can function as transcriptional regulatory domains to modulate gene expression profiles^11–13^. One TF which has been shown to bind TEs is the CCCTC-binding factor (CTCF)^14–16^. This zinc finger protein has a well-established role in modifying the complex 3D entity of the human genome by the formation of topologically associated domains (TADs), sub-megabase regions which can form intradomain interactions, generating loops between regulatory regions (enhancers and promoters)^17,18^. These interactions can have an impact on gene expression over far-reaching genomic distances (> 100,000 kb)^14^. The complexity of SVA directed gene expression dynamics is compounded by SVA polymorphisms which occur in two major forms: sequence variation within the SVA itself (especially the CT hexamer repeat and VNTR) and secondly polymorphism for its presence or absence with respect to the reference human genome, which is also referred to as retrotransposon insertion polymorphism (RIP)^9,19^. One well-characterised SVA RIP which causes X-linked dystonia parkinsonism (XDP) is found in intron 32 of the TATA-box binding protein associated factor 1 (*TAF1*) gene^20^. This insertion not only led to reduced *TAF1* mRNA expression by intron retention, but additionally, variation within the CT hexamer repeat (ranging between 35 and 52 repeats) of this class F SVA inversely correlated with age at onset (AAO) of the disease^20–22^. To date, at least 24 disease-causing SVA insertions were reported which highlights the impact of SVA elements on gene function and genetic processing ultimately leading to pathogenesis and phenotypic differences within a population^13,23,24^.

**Figure 1:**
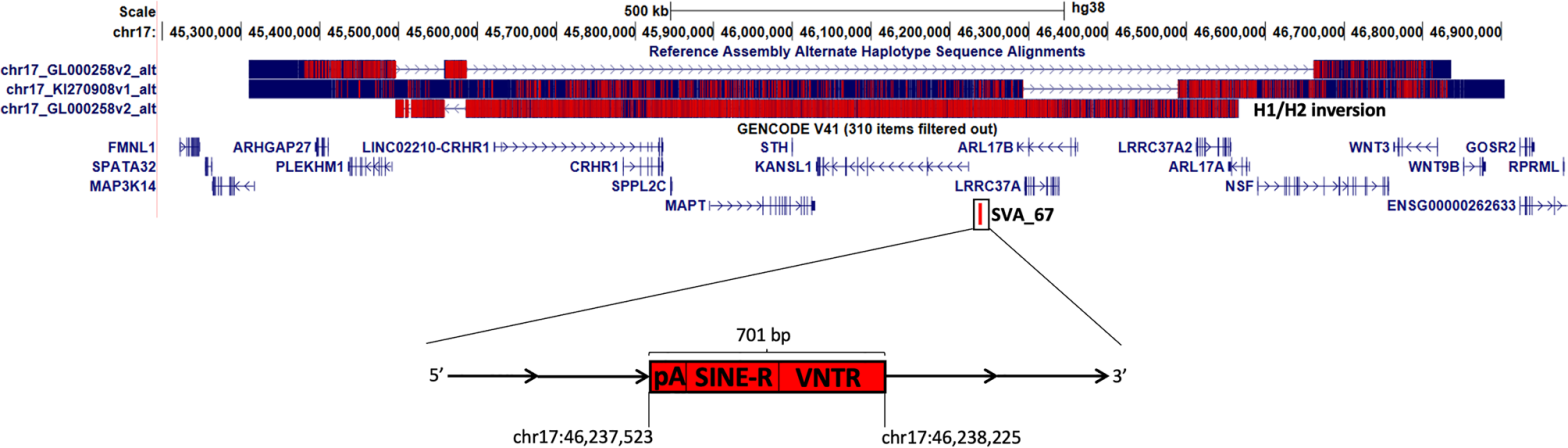
Schematic representation of the *MAPT* locus on chromosome 17 modified from UCSC genome browser hg38. This locus contains a complicated inversion polymorphism called *H1*/*H2*. *H1* represents the canonical sequence and while *H2* is characterised by a ~ 1 Mb inversion (visualised at the top). The presence of these haplotypes makes it difficult to describe other regulatory elements in this region. SVA_67 (chr17:46,237,519–46,238,226) (red bar at the bottom) represents a truncated SVA element (701 bp) and is located approximately 12 kb upstream and in sense orientation with reference to the 5’ transcriptional start site of *KANSL1*. This SVA is present in the *H1* haplotype and absent in *H2*. Start and end coordinates of SVA_67 are shown.

The specific SVA which is the focus of this study, termed SVA_67 (chr17:46,237,519–46,238,226), is at the microtubule-associated protein tau (*MAPT*) locus on chromosome 17q21. This locus is characterised by a ~ 1Mb inversion (chr17:45,495,831–46,565,079) featuring two predominant haplotypes (*H1* and *H2*), whereby *H2* represents the inverted sequence relative to the common human reference sequence of *H1*^25–28^ (Fig. 1). The inversion frequency differs across populations and is primarily found in the Mediterranean with ranges within Europe between 5 and 37.5%^26^. Parkinson’s disease (PD), frontotemporal dementia (FTD) and progressive supranuclear palsy (PSP) are just a few neurodegenerative diseases that have been associated with the *H1* haplotype^28–32^. When looking at PD, previous genome-wide association studies (GWAS) have shown that the major haplotype *H1* is genetically associated with this condition^31–36^.

In our previous studies, we characterised the impact of SVA RIPs using transcriptomic, whole genome sequencing and clinical data from the Parkinson’s Progression Markers Initiative (PPMI) cohort highlighting the association of seven RIPs with the progression of PD and with differential gene expression^37^. Moreover, our recent study also showed the association of the presence or absence of a particular SVA RIP, termed SVA_67, with differential isoform expression of several genes within the *MAPT* locus^38^. SVA_67, located about 12 kb upstream of the KAT8 regulatory NSL complex subunit 1 (*KANSL1*) gene, represents a 5´ truncated SVA element with a length of 701 bp (hg38) and tags *H1* while its absence is associated with *H2*^37,38^ (Fig. 1). It is, therefore, a challenge defining regulatory associations within the large genomic inversion. Nevertheless, differential gene expression at the *MAPT* locus which can be predominantly explained by the *H1*/*H2* haplotype inversion^39^ is modified by polymorphism within SVA_67.

In this study, we demonstrate SVA_67 specific regulation of gene expression across the genome. We show that SVA_67 can act as an expression quantitative trait loci (eQTL) not only locally at the *MAPT* locus but also over large distances and across chromosomes. We identified a polymorphism within the primary sequence of SVA_67 in the form of four different alleles of its VNTR with a size difference of 357 bp between the smallest (allele 1) and largest (allele 4) domain which demonstrated both differential properties of a regulatory domain in a reporter gene assay and correlation with differential expression of multiple genes using transcriptomic datasets from the PPMI cohort. Interestingly, SVA_67 alleles were associated with multiple PD progression markers (including caudate symmetry index) which was also supported by a trend of the VNTR to correlate with PD AAO. These findings increase our understanding of SVAs not only as contributors to interpersonal gene expression patterns in the population but also raise awareness of their contribution to the missing heritability of neurodegenerative diseases.

## Results

### SVA_67 demonstrates eQTL effect in *cis* and *trans*

**Figure 2:**
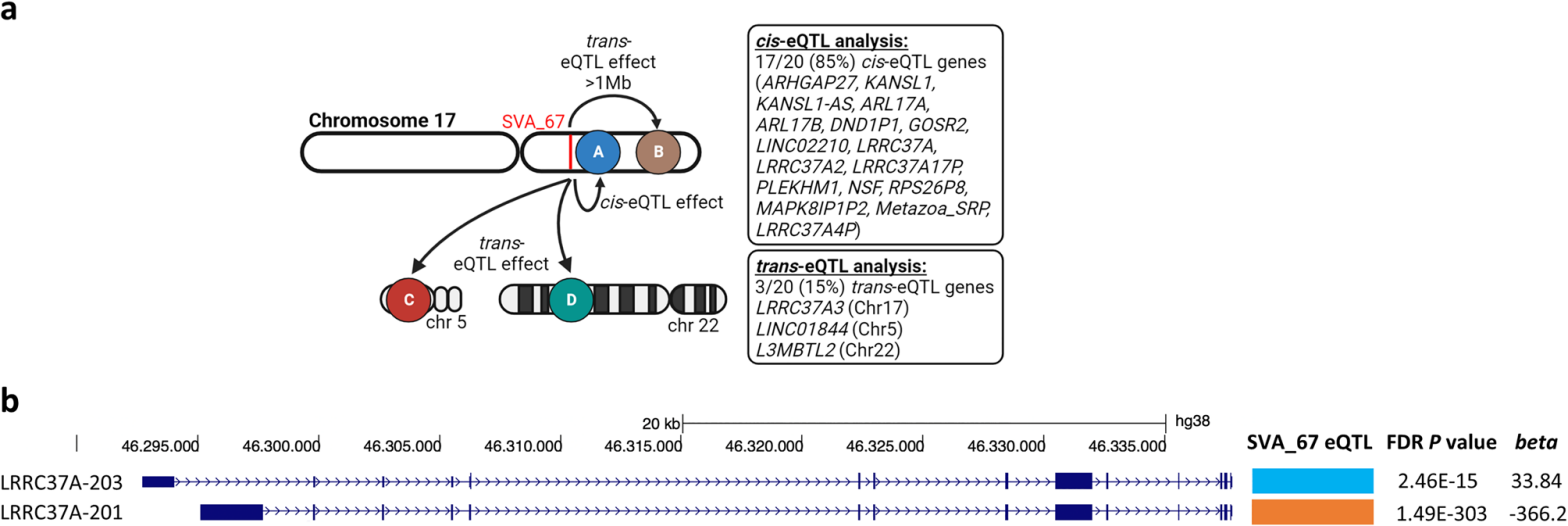
SVA_67 exhibits eQTL effect in *cis* and *trans*. (**a**) Schematic representation of the effect of SVA_67 (red bar) across the genome. Genes that are affected in *cis* and *trans* are indicated. (**b**) SVA_67 shows opposite effects on different isoforms of the same gene (here *LRRC37A*). Blue block represents activating and orange block repressive effect. Genome-wide FDR corrected *P* values and *beta* value are indicated. LRRC37A-201 (ENST00000320254), LRRC37A-203 (ENST00000496930).

A combination of whole genome and blood-derived transcriptomic sequencing data from the PPMI cohort was used to perform eQTL analysis on an isoform-based level. This analysis demonstrated that SVA_67 modulates the expression of multiple genetic loci at a genome-wide significance level (FDR) below 0.05. To define the magnitude of the effect we set up the effect size represented by the *beta* value (slope coefficient). We identified 74 isoforms derived from 20 genes (17 *cis*-eQTL and 3 *trans*-eQTL genes) that were differentially regulated by SVA_67 analysed as a RIP (Fig. 2A**, **Supplementary Data 1). Of these isoforms, 38 were up-regulated and 36 down-regulated. More locally, SVA_67 represents an intergenic variant located 12 kb downstream and 54 kb upstream of the *KANSL1* and *LRRC37A* gene, respectively, relative to their major transcriptional start site. When addressing *LRRC37A*, SVA_67 had a statistically significant eQTL effect for two *LRRC37A* isoforms, exhibiting both positive or negative effects which were isoform specific. SVA_67 showed a strong repressive effect on isoform LRRC37A-201 (*beta* = − 366.2; *P* = 1.49E–303) while an activating effect was observed for LRRC37A-203 (*beta* = 33.84, *P* = 2.46E–15) (Fig. 2B). The highest *beta* value for *KANSL1* was 239 (KANSL1-204 isoform, *P* = 1.84E–113) and the lowest − 594 (KANSL1-225 isoform* P* = 5.12E–293). Other genes affected by SVA_67 include *ARHGAP27*, *ARL17A*, *ARL17B*, *PLEKHM1*, and *NSF* among others with a *P* value up to 10E-303, and maximal and minimal *beta* values of 468 and − 160, respectively (Supplementary Data 1). In addition to this *cis* effect, SVA_67 also had the ability to affect isoform expression in *trans* (threshold > 1M bp), with 11 isoforms derived from 3 genes (*LRRC37A3*, *L3MBTL2* and *LINC01844*) being trans-regulated indicating the plethora of distinct mechanisms of SVA_67 as an eQTL (Fig. 2A).

### SVA_67 is polymorphic for its VNTR domain

**Figure 3:**
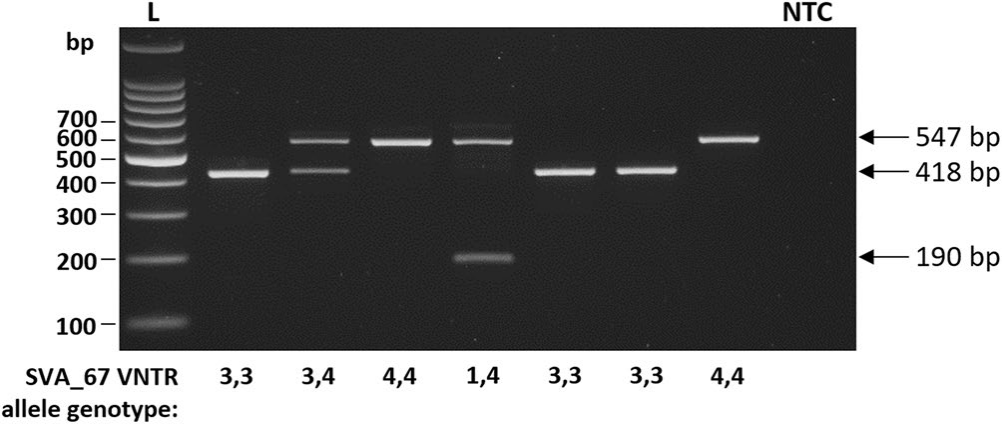
SVA_67 is polymorphic for its VNTR domain. Genotyping PCR and gel electrophoresis of SVA_67 from a subset of the PPMI cohort at the *MAPT* locus. 91 PPMI samples were used for genotyping of SVA_67 using a primer set amplifying over the VNTR domain. Representative gel image with expected amplicon size for SVA_67 alleles and corresponding SVA_67 genotypes are shown. Four SVA_67 alleles were identified (numbered 1–4 to reflect increasing length). Allele 2 was excluded from downstream analyses. *NTC* non-template control. Figure shows a cropped image. Uncropped gel can be found in Supplementary Fig. 4, Supplementary Data 4.

To assess polymorphism within the VNTR domain of SVA_67, genotyping using nested PCR was performed in 91 individuals characterised by the SVA_67 containing *H1*/*H1* haplotype from the PPMI cohort. In this PCR, the product of a first reaction (amplifying SVA_67 with flanking sequences) was used as the template for the second reaction placing one primer in the flanking sequence of SVA_67 and a second primer internal to the SVA, allowing the specific amplification over the VNTR domain. Primary sequence polymorphism within the VNTR domain of SVA_67 was identified, with four alleles (´1´, ´2´, ´3´, ´4´) of the VNTR found (Fig. 3). The size difference between the shortest (termed ´1´) and longest (termed ´4´) VNTR differed in length by 357 bp (Fig. 3, Supplementary Figs. 1 and 2). SVA_67 VNTR allele 2 was excluded from downstream analyses (tagging SNP, reporter gene assay and expression analyses) due to an allele frequency of 0.55% and therefore limited power. To increase sample size, tagging SNPs for the three most common alleles (1, 3, 4) were generated. Computational prediction showed 92% identity with previously PCR genotyped SVA_67 alleles and were therefore taken forward for downstream analyses. Genotypes for 295 individuals (97 controls (CO), 198 PD) were predicted within the PPMI cohort. Allele 3 represented the most common allele with an allele frequency of 46% in CO and 44% in PD individuals, respectively, followed by allele 4 (35% CO, 37% PD) and allele 1 (19% CO, 18% PD) (Table 1). Statistical analysis between case and control showed no difference in genotype or allele frequency (Table 1).

**Table 1:** SVA_67 VNTR genotype and allele frequencies in the PPMI cohort and stratification by CO and PD individuals.

	Frequency		Percentage		*P* value (Fisher’s exact test)
	Control	PD	Control	PD	
Genotype					
1, 1	5	7	5%	4%	0.54
1, 3	14	37	14%	19%	0.18
1, 4	12	22	12%	11%	0.85
3, 3	21	39	22%	20%	0.76
3, 4	34	60	35%	30%	0.43
4, 4	11	33	11%	17%	0.30
Total	97	198	100%	100%	
Allele					
1	36	73	19%	18%	1.00
3	90	175	46%	44%	0.66
4	68	148	35%	37%	0.65
Total	194	396	100%	100%	

Haploview was used to predict genotype frequencies in 295 individuals (97 CO, 198 PD). Fisher’s exact test with a 95% confidence interval was used for statistical analysis of genotype and allele frequencies between cases and controls.

### SVA_67 alleles are functionally repressive in a luciferase reporter gene assay

**Figure 4:**
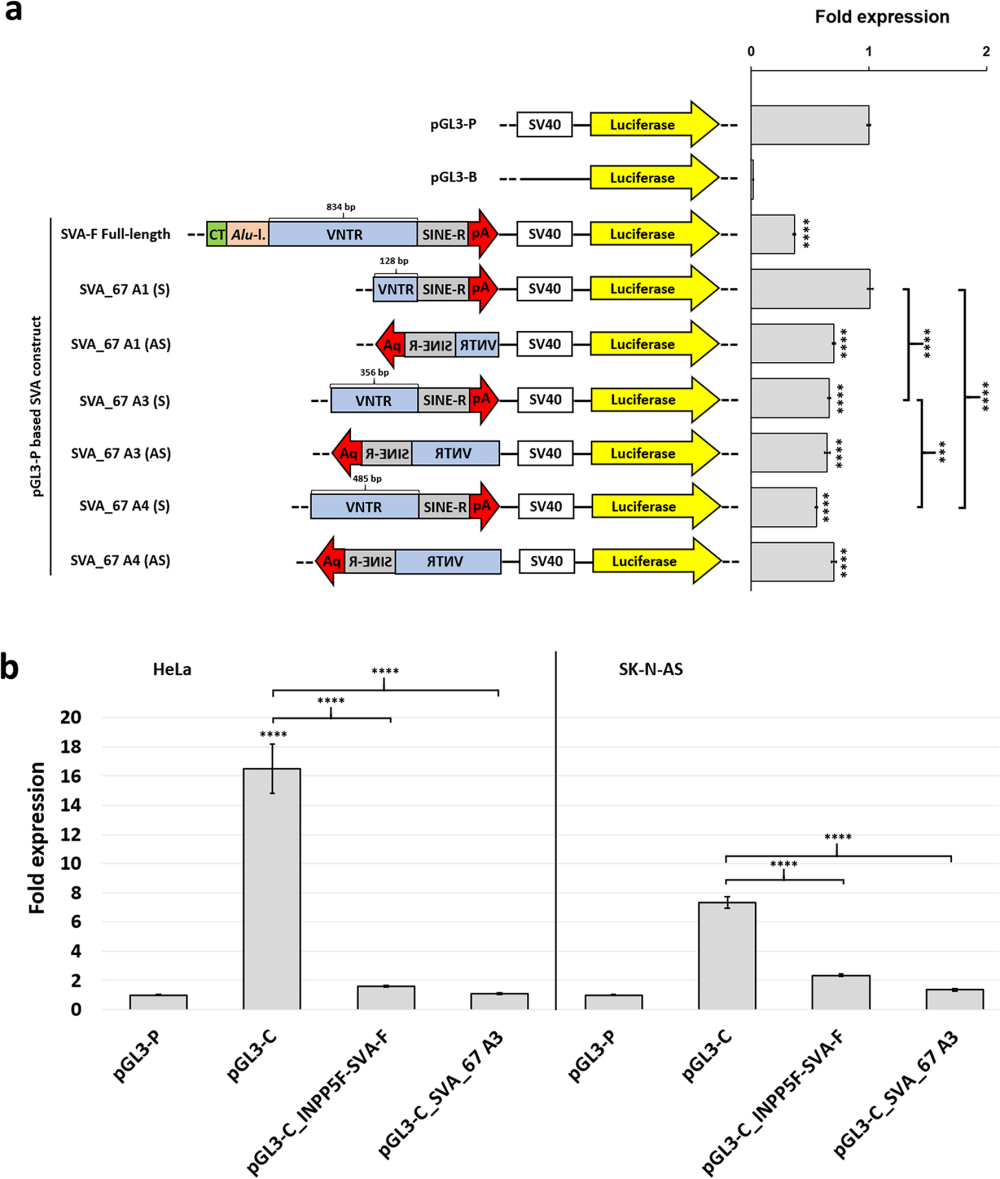
SVA_67 alleles demonstrate repressive functional properties in luciferase reporter gene assays. (**a**) SVA_67 alleles 1, 3 and 4 were tested in sense (S) and anti-sense (AS) orientation relative to the SV40 promoter located upstream of the luciferase reporter gene in HEK293 cells. The vector pGL3-B does not contain a promoter and represents the negative control. As an additional control, a full-length SVA-F element (derived from the *INPP5F* locus) containing the five key domains (CT element, *Alu*-like region, VNTR, SINE-R and poly A) was included. Schematic for pGL3-Promoter (pGL3-P), pGL3-Basic (pGL3-B) and SVA_67 allele-containing constructs with corresponding VNTR lengths are shown. (**b**) To further characterise the repressive effect, we cloned the most common allele 3 and the full-length SVA-F (from *INPP5F* locus) upstream of the SV40 promoter into the pGL3-Control (C) vector which contains an enhancer element providing high level reporter gene expression in HeLa and SK-N-AS cells. All data is normalised to the pRL-TK renilla control vector signal and displayed as fold change to pGL3-P (= onefold). Biological replicates n = 3 with technical replicates within each assay n = 3. The mean ratios of each experiment were compared using ANOVA with Bonferroni correction for multiple comparisons to calculate statistical significance between pGL3-P, pGL3-C and SVA_67 allele-containing pGL3-P/-C constructs, respectively. ***p < 0.001; ****p < 0.0001. Bars, arithmetic means ± s.e.m. of technical replicates. A1, Allele 1; A3, Allele 3; A4, Allele 4.

To assess the regulatory roles of the SVA_67 VNTR alleles in vitro, luciferase-based reporter gene constructs were generated. For this, the three most common SVA_67 alleles (1, 3, 4) were cloned upstream of the SV40 minimal promoter of pGL3-Promoter (P) based vectors in sense and anti-sense orientation. Luciferase activity of generated SVA_67 constructs was compared to the activity of pGL3-P vector alone (baseline expression) and a full-length SVA-F element derived from the *INPP5F* locus in HEK293 cells (Fig. 4a). Both full-length SVA-F and truncated SVA_67 alleles showed repressive characteristics compared to baseline expression (*P* < 1.00E-04). The full-length SVA-F element significantly repressing (0.37, *P* < 1.00E–04) luciferase activity compared to baseline expression (empty pGL3-P, 1.00) (Fig. 4a). SVA_67 alleles also showed significant repressive effects on reporter gene expression. To validate the action as a repressor we cloned the most common SVA_67 allele 3 and the full-length INPP5F-SVA into pGL3-Control (C). The pGL3-C construct was chosen as it contains the SV40 enhancer, providing high reporter gene expression compared to the pGL3-P construct which lacks this enhancer. Using HeLa and SK-N-AS cell lines, pGL3-C empty vector led to changes in reporter gene activity up to 16.5- (HeLa) and 7.3-fold (SK-N-AS), respectively, compared to pGL3-P (Fig. 4b). When containing the truncated SVA_67 and full-length INPP5F-SVA, respectively, upstream of the SV40 promoter, reporter gene activity was significantly (*P* < 1.00E–04) reduced and the enhancer activity largely abolished, thereby displaying a similar effect between both SVA constructs (Fig. 4b). No major difference between pGL3-C_SVA_67 and pGL3-C_INPP5F-SVA was obtained suggesting a common repressor interacting with both full-length and truncated SVA element.

### SVA_67 VNTR alleles significantly correlate with differential gene expression for a number of genes

**Figure 5:**
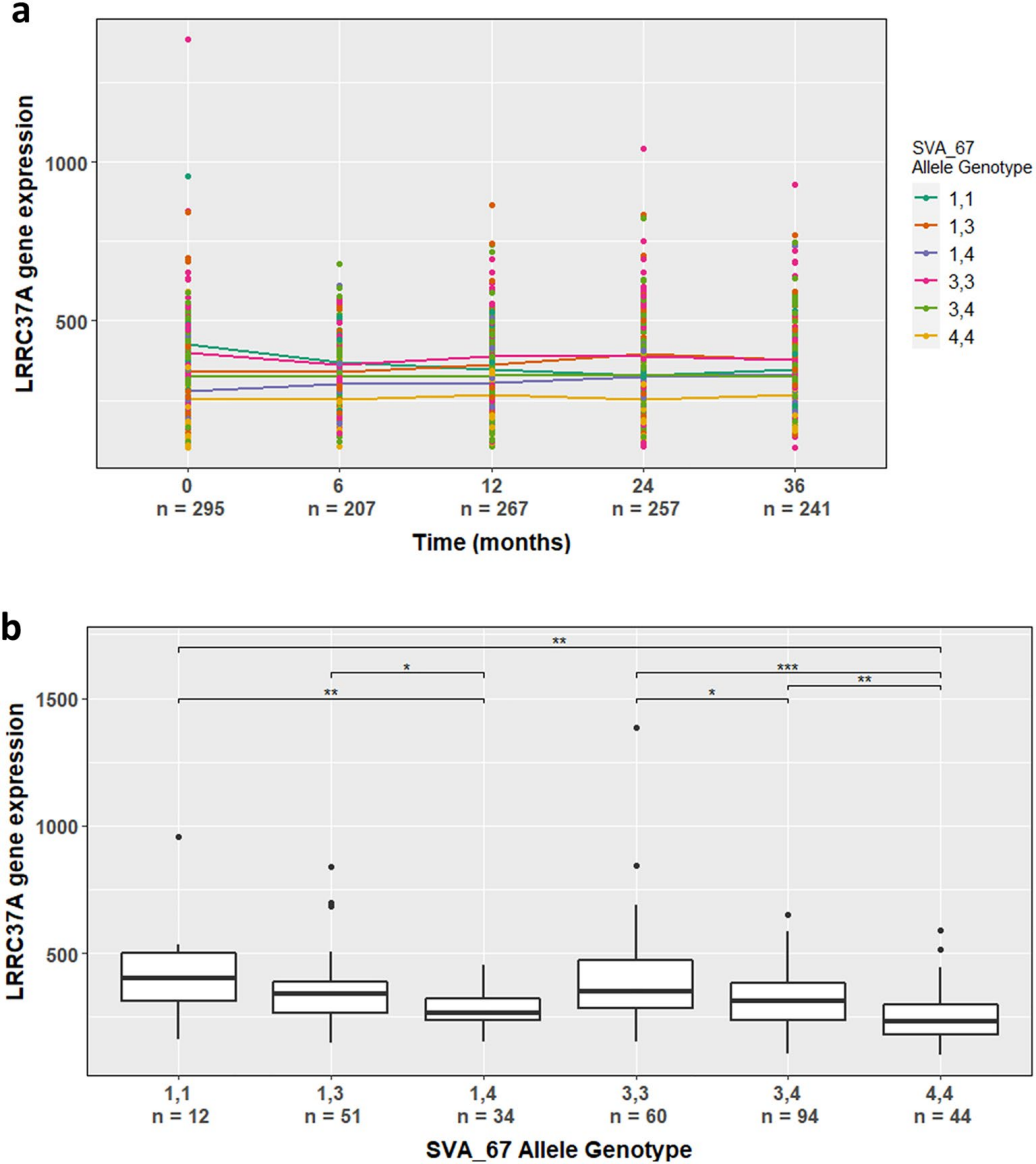
SVA_67 VNTR allele genotype is significantly associated with differential *LRRC37A* gene expression. (**a**) Using the PPMI cohort, datasets of individuals from up to five visits were available (baseline/0 months; 6 months; 12 months; 24 months; 36 months). *LRRC37A* gene expression was stratified by SVA_67 allele genotype and visit. *LRRC37A* expression levels varied stratified by VNTR genotype (*P* < 2.2E–16) but did not change over the time course (*P* = 8.77E-01). (**b**) Association of SVA_67 allele genotype with *LRRC37A* gene expression using datapoints from Baseline (0 months). Statistically significant differences between SVA_67 allele genotypes and visits were calculated using the non-parametric Kruskal–Wallis test. The Wilcoxon test was applied to calculate pairwise comparisons between group levels with corrections for multiple testing (FDR correction) indicated as asterisks. *P < 0.05, **P < 0.01, ***P < 0.001.

To assess the differential influence of SVA_67 primary sequence polymorphism on gene expression, we correlated the corresponding VNTR genotypes with transcriptomic datasets obtained from the PPMI cohort (Table 2). For analysis, we looked at eight genes (*KANSL1*, *LRRC37A*, *PLEKHM1*, *MAPT*, *CRHR1*, *ARL17A*, *ARL17B* and *NSF*) located inside and outside the *H1*/*H2* polymorphism (Fig. 1). We first analysed SVA_67 associated expression levels between baseline (BL/V0) and four visits indicated by months after BL (6 months/V02, 12 months/V04, 24 months/V06, 36 months/V08). For analysis, we combined available data from CO and PD individuals similar to our previous studies on the SVA_67 RIP^32,33^. When addressing *LRRC37A*, expression levels varied stratified by VNTR genotype (*P* < 2.2E-16), but did not change over the time course (*P* = 8.77E-01) (Fig. 5a). The same trend was visible for *KANSL1*, *ARL17A*, *ARL17B* and *NSF*, while the genes *CRHR1*, *MAPT* and *PLEKHM1* showed expression changes over the time course (Supplemental Data 2). We then assessed differential gene expression using BL data to compare actual levels of expression. We demonstrated that differences in SVA_67 VNTR sequence are significantly associated with the differential expression of 6 of these 8 genes, while the expression of *PLEKHM1* and *CRHR1* was not affected (Supplementary Fig. 3b,d, Supplemental Data 2). We noted that SVA_67 allele genotype 3,3 was significantly associated with changes in *MAPT* expression compared to genotype 1,3 and 1,4 (FDR *P* = 3.77E-02) (Supplementary Fig. 3c). However, due to the low *MAPT* expression levels, these results should be interpreted with caution. For *LRRC37A*, individuals with 3,3 genotypes show a 19.2% and 36.7% higher *LRRC37A* expression compared to individuals with 3,4 (FDR *P* = 1.32E–02) and 4,4 (FDR *P* = 8.20E–06) genotypes, respectively (Fig. 5b). The repressive effect of allele 4 is also seen in association with allele 1. Individuals carrying the 1,4 and 4,4 SVA_67 allele genotype show 34.5% (FDR *P* = 9.68E–03) and 40.8% (FDR *P* = 3.33E–03) lower *LRRC37A* expression to individuals containing two copies of the shortest allele 1 (Fig. 5b). This characteristic repressive effect of allele 4 was also seen for *KANSL1*, *ARL17A* and *ARL17B*. (Supplementary Fig. 3a,e,f). Interestingly, the gene *NSF* showed an opposite effect; allele 3 represented the most repressive allele, individuals containing two copies of allele 3 (3,3 genotype) displaying a 61% (FDR *P* = 7.30E–07) and 22.6% (FDR *P* = 6.60E–03) reduction in expression compared to 1,1 and 4,4 genotypes (Supplementary Fig. 3g).

**Table 2:** Demographics of the PPMI subjects used in this study.

Variable	PPMI individuals	
	PD (n = 198)	CO (n = 97)
Sex		
Male	129 (65%)	64 (66%)
Female	69 (35%)	33 (34%)
Age		
Mean (min, max)	61.3 (33.5–84.9)	60.7 (32.2–82.7)

**Figure 6:**
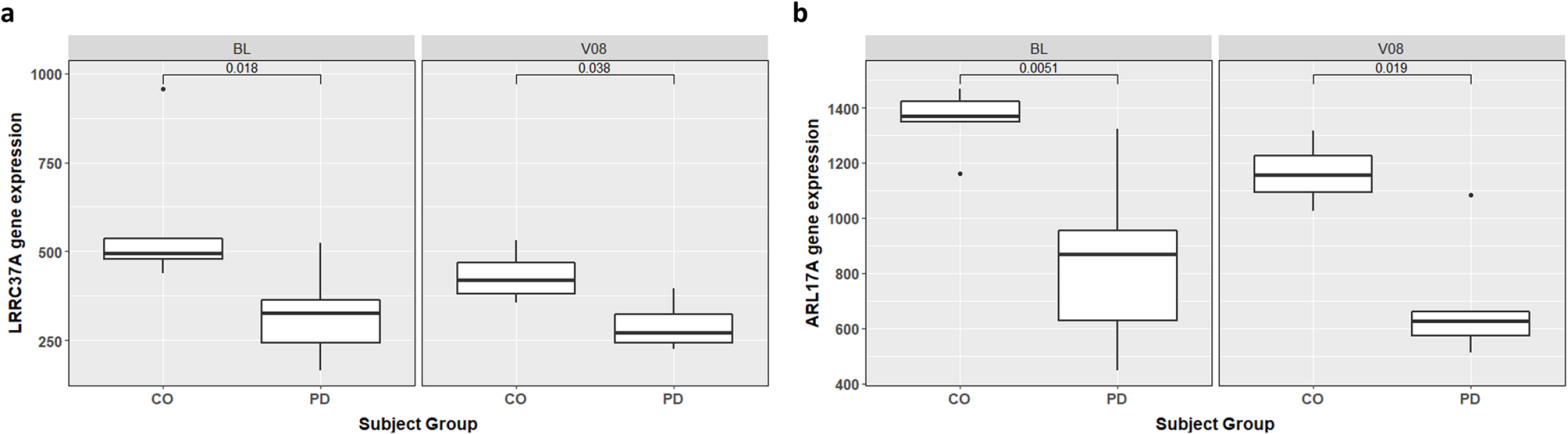
SVA_67 VNTR allele 1 correlates with reduced gene expression in PD individuals. *LRRC37A* (**a**) and *ARL17A* (**b**) gene expression is significantly reduced in PD patients carrying the 1,1 allele genotype. Gene expression was stratified by SVA_67 allele genotype and individual group (CO vs PD) using datapoints from BL (0 months) and V08 (36 months). The Wilcoxon test was applied to calculate pairwise comparisons between individual group levels. CO, control individual; PD, Parkinson’s disease individual. BL (n = 5 (CO), n = 7 (PD)); V08 (n = 4 (CO), n = 6 (PD)).

Furthermore, we assessed gene expression changes by individual type (PD vs CO) at BL and 36 months (V08) later. Interestingly, PD individuals homozygous for the shortest SVA_67 allele 1 (genotype 1,1) show a significant reduction of *LRRC37A* and *ARL17A* expression, with 45.3% (*P* = 1.80E–02) and 38.9% (*P* = 5.10E–03) compared to controls (Fig. 6a,b). This expression remained reduced in PD patients 36 months (V08) after BL, with a decrease of 32.9% (*P* = 3.80E–02) for *LRRC37A* and 41.7% (*P* = 1.90E–02) for *ARL17A* compared to controls (Fig. 6a,b).

### SVA_67 VNTR alleles correlate with PD progression markers and reduced gene expression in PD individuals

**Figure 7:**
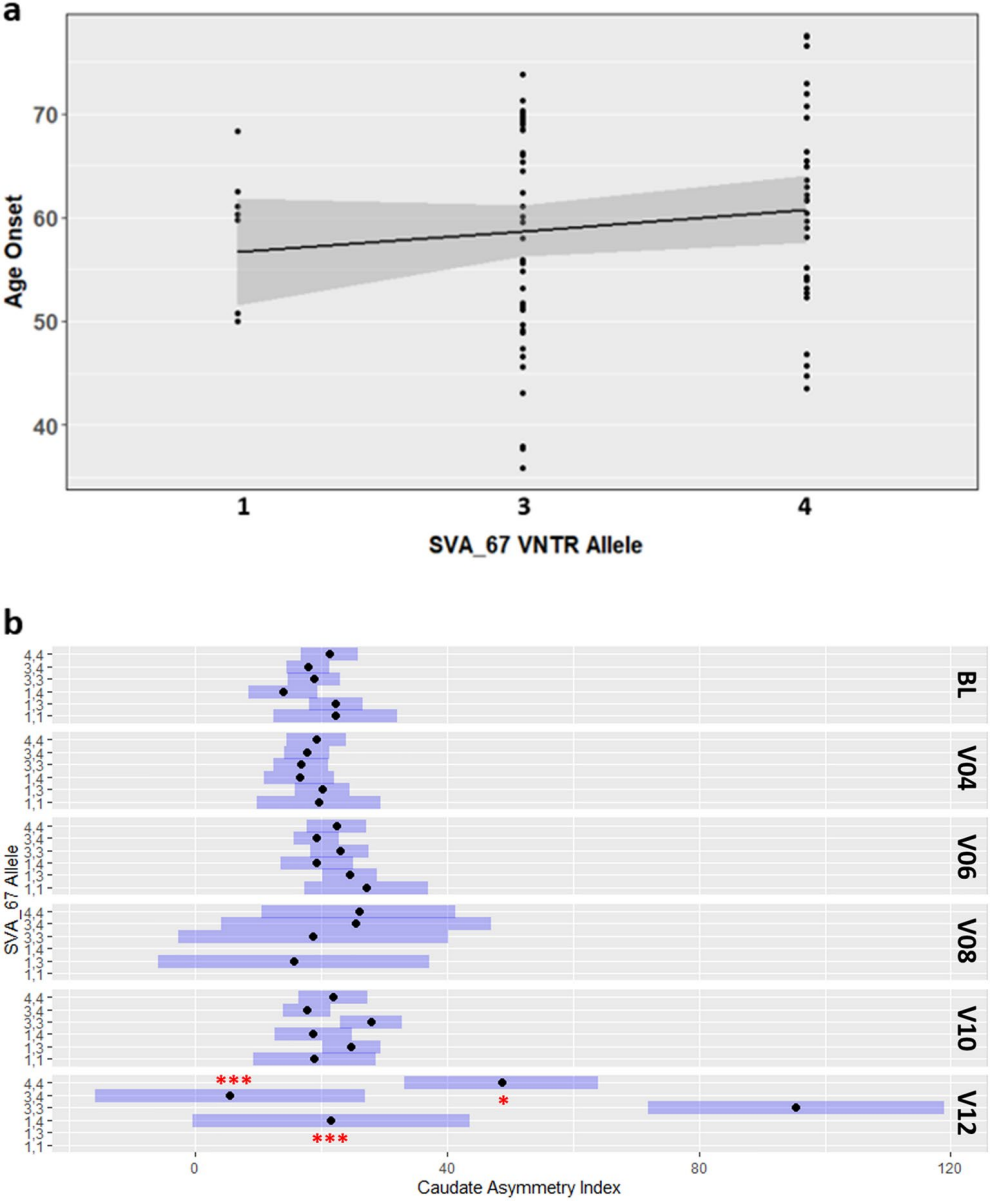
SVA_67 VNTR alleles correlate with PD progression markers in the PPMI cohort. (**a**) Length of SVA_67 VNTR correlates directly with PD age at onset. Linear regression analysis (n = 79, *R*^*2*^ = 0.004839, *P* = 2.44E-01) was performed using three different alleles of SVA_67 (1, 3, 4) characterised by different lengths of the VNTR domain. 1 (n = 7), 3 (n = 39), 4 (n = 33). (**b**) SVA_67 allele genotype significantly correlates with differential caudate asymmetry index (CAI). Allele genotype 3,3 shows higher CAI compared to 1,4, 3,4 and 4,4 genotypes. 95% confidence intervals are represented by the blue bar. *P < 0.05, ***P < 0.001 (Tukey adjusted *P* values are reported compared to 3,3 genotype).

We had previously associated the presence/absence SVA_67 polymorphism with PD progression, therefore we wanted to address whether there were PD-associated parameters with the SVA primary sequence polymorphism. As the primary sequence in the XDP disease causing SVA was associated with age at onset^20^ we addressed if a similar association occurred for SVA_67. As SVA_67 represents a truncated SVA element with the CT element missing (Fig. 1) we focused on the variation within the VNTR domain. We performed linear regression to determine the association of age at onset and VNTR length, which demonstrated that the longest VNTR domain (allele 4) correlated with a higher age at onset compared to the other variants (Fig. 7a), however this did not reach statistical significance (*R*^2^ = 0.004839, *P* = 2.44E–01). Due to the availability of clinical and phenotypic data from the PPMI cohort, we correlated SVA_67 alleles with 114 PD progression markers. We show that SVA_67 is significantly associated with a number of progression markers including primary diagnosis (*P* = 4.54E–05), Modified Schwab & England ADL Score (*P* = 1.23E–02), SCOPA-AUT Sexual Dysfunction Score (*P* = 1.26E–02), Hoehn & Yahr Stage (*P* = 3.49E–02), ratio of CSF p- and t-tau to CSF A-beta 1–42 (*P* = 4.01E–02 for p-tau and *P* = 4.93E–02 for t-tau) and caudate asymmetry index (*P* = 2.00E–03) (Supplemental Data 3). This caudate asymmetry index (CAI) was derived from DaTscan single photon emission computed tomography (SPECT) imaging. We demonstrated that SVA_67 allele genotype 3,3 showed significantly higher CAI compared to genotypes 3,4 (*P* < 1.00E–04), 4,4 (*P* = 1.44E–02) and 1,4 (*P* = 1.00E–04) at V12 (Fig. 7b). In addition, individuals with 4,4 SVA_67 allele genotype show increased CAI compared to individuals with 3,4 genotypes (*P* = 1.76E–02).

## Discussion

Our study expanded the impact of SVA_67 as a regulatory domain within the *MAPT* locus, a key region in the genome associated with neurodegeneration including PD, by exploring the role of its polymorphism as both a transcriptional regulatory domain and biomarker for PD progression. We show that SVA_67 is polymorphic for its VNTR primary sequence (Fig. 3, Supplementary Fig. 2), containing a minimum of four variants in addition to the previously described RIP for presence/absence in the genome^37,38^. The *MAPT* locus is contained within a large genomic inversion termed *H1*/*H2*. Although the majority of studies to date address genetic correlations for neurodegeneration with the inversion, we have here and in previous studies highlighted the role of a hominid SVA RIP in the inversion, termed SVA_67, to modulate PD progression and gene expression. In this communication, by focusing on primary sequence variation within the SVA itself we demonstrate by combining whole genome sequencing and transcriptomic data from the PPMI cohort that SVA_67 represents an eQTL in *cis* and *trans*, thereby not only being restricted to local changes proximal to its position within the *MAPT* locus but also genome-wide (Fig. 2). We highlight clinical significance of such VNTR polymorphism by showing a significant association with several PD progression markers and a correlation with the age of onset of PD, although the latter did not reach statistical significance, potentially due to the power of the study or that it is one component of a polygenic risk for AAO (Fig. 6). One of these progression markers represented the caudate asymmetry index (CAI). This data is derived from DaTscan SPECT analysis which involves dopamine transporter (DAT) imaging assessments, whereby a reduction of striatal DAT is not only characteristic for PD but can also be applied to follow dopaminergic degeneration^40,41^. SVA_67 allele genotype 3,3 showed a significant association with CAI (Fig. 7) which may suggest that this polymorphism may be associated in modifications to PD course. Intriguingly, PD patients carrying two copies of the shortest SVA_67 allele 1 had significantly reduced expression of two genes, one of which was *LRRC37A* (Fig. 6).

The SVA_67 polymorphism can differentially modulate the transcriptome both locally and globally. Previous work has shown that SVA retrotransposons more generally have the ability to modulate gene expression in vitro and in vivo^11,12^. Our data on the action of SVA_67 acting as an eQTL is consistent with a study by Wang et al*.*, which incorporated TE polymorphism with gene expression using datasets from the 1000 Genome Project and showed that many TE RIPS had the ability to act as eQTL, thereby showing a population- and cell-type-specific transcriptomic influence^42^. We demonstrate that SVA_67 had the ability to affect many loci (20) ranging from genes located nearby (e.g. *KANSL1* and *LRRC37A*) and across chromosomes (e.g. *L3MBTL2* on chr22) (Fig. 2). As part of the nonspecific lethal complex, *KANSL1* was previously identified as an essential gene for autophagy, a key machinery to maintain proteostasis, and dysregulation has been considered to play major roles in neurodegenerative diseases including amyotrophic lateral sclerosis and PD^43–45^. In addition, *KANSL1* is also involved in the regulation of PINK-1-regulated mitophagy which has been shown to be dysregulated in a subset of familiar PD cases^46,47^. A key feature of our eQTL analysis represented the possibility to assess the influence of SVA_67 on expression on an isoform-based level. Interestingly, SVA_67 showed opposite effects (activating/repressing) on different isoforms of the same gene, as shown for the isoforms LRRC37A-201 and LRRC37A-202 of the *LRRC37A* gene (Fig. 2). Bowles et al*.* recently used post-mortem RNA-seq data and identified *LRRC37A* as a novel gene within the *MAPT* locus to be associated with PD via both its interplay with α-synuclein and its role in inflammation^48^. Differential gene expression directed by SVA_67 could be one of the mechanisms involved with PD risk and progression. Imbalanced expression of one gene or a combination of multiple genes within the *MAPT* locus involved in key disease pathways (e.g. intracellular trafficking, impaired autophagy) could contribute to disease progression. SVA_67 could play a key role by being a genetic variant responsible for the effects seen which involves the modulation of transcription. These regulatory effects from SVA_67 could be small and numerous, however, numerous small effects over long time periods could modulate complex diseases, such as PD, due to a cumulative effect of multiple low-contribution variants.

As an initial analysis to determine the functional aspects of SVA_67 to act as a regulatory domain we addressed its ability and that of the VNTR variants in reporter gene constructs in vitro. Reporter gene assays demonstrated a strong repressive effect of SVA_67 VNTR alleles. Interestingly, when using enhancer containing pGL3-C vectors, this repressive effect was similar compared to a full-length SVA element (Fig. 4). This would suggest the involvement of a common repressor mechanism between both the full-length and truncated SVA_67 element. Future experiments can validate these results in brain relevant cell models such as patient-derived induced pluripotent stem cells with known hallmarks of PD (known mutations etc.) and neuronal differentiations. This model shown here can be used to gain insight into gene regulatory mechanisms in a ‘test tube’ approach. Variation within SVA_67 VNTR domain itself also had the ability to differentially affect the expression of multiple genes at the *MAPT* locus in vivo (Fig. 5). VNTRs and repeat expansions are of special interest as they have been heavily studied as functional variants and biomarkers in neurodegenerative diseases, as reported for the *C9orf72* hexanucleotide and ataxin-1 and -2 repeat expansions associated with ALS and a VNTR within the ALS risk gene *CFAP410*^40,41,49,50^*.* As mentioned before, one well-characterised SVA polymorphism in the form of the variation of the CT hexamer repeat is inversely correlated with XDP age of onset^20–22^.

The *H1* inversion is associated with an increased risk of developing PD, however, the genetic variant(s) responsible for this risk remains unclear. We previously showed the association of SVA_67 presence/absence with differential gene and isoform expression of several genes in the *MAPT* locus, however, as SVA_67 is present in *H1* and absent in *H2*, this effect cannot be fully differentiated from the haplotype specific influence on gene expression^37,38^. A study by O’Brien et al. combined genome-wide genotyping with transcriptomic data and showed that differential expression of 13/21 genes at the *MAPT* locus can be “largely explained” by the *H1*/*H2* inversion status^39^. However, the involvement and impact of other regulatory variants, e.g. in form of TEs, has yet to be established. Our data suggests that polymorphism within SVA_67 is one of the elements which needs to be incorporated into our understanding of the role of *H1* in neurodegeneration. In our recent study we validated the influence of SVA_67 insertion polymorphism on gene expression profiles in an otherwise identical genetic background by CRISPR technologies^51^. These results highlighted that SVA_67 had the capacity to function as a modulatory domain at the *MAPT* locus by significantly modulating gene transcriptomic profiles^51^. We cannot yet define disease-risk alleles, however we show that polymorphisms within SVA_67 are associated with PD progression markers and a trend for age at onset demonstrating how such elements might harbour some of the missing heritability associated with neurodegeneration. Our study focused on a single genetic variant, SVA_67, which we demonstrate can modulate a plethora of transcriptomic changes globally. This analysis benefited from the use of valuable longitudinal data from the PPMI cohort. While our study provides valuable insights into the association between SVA_67 polymorphism and PD, several limitations should be acknowledged. Firstly, it is important to note that only around 10% of PD cases in Caucasians are currently explained by clear genetic causes^52–54^, indicating the complex and multifactorial nature of PD etiology. The association between the *H1*/*H2* inversion polymorphism and PD susceptibility is primarily based on GWAS, and further investigations are needed to confirm and elucidate the underlying mechanisms of this association. Future studies incorporating larger and more diverse cohorts, as well as functional characterisation of the *H1*/*H2* inversion polymorphism as well as TE variations, are needed to validate their role in PD pathogenesis and identify potential therapeutic targets. This could lead to a more detailed understanding of SVAs not only as contributors to interpersonal gene expression patterns in the population but could also raise awareness of the involvement of transposable elements in the missing heritability of neurodegenerative diseases.

## Materials and methods

### PPMI cohort used for SVA_67 VNTR genotyping and analysis of differential gene expression

As part of this study, genomic DNA (gDNA) preparations and transcriptomic data from the PPMI cohort (www.ppmi-info.org) were used (Table 2). This cohort contains longitudinal data with the aim to follow PD patients and to better describe the disease course. In addition to transcriptomic data from the blood, this cohort also contains genetic and clinical data. Transcriptomic data from four visits (baseline, BL (at diagnosis, V0), and after follow-up of 6 months (V02), 12 months (V04), 24 months (V06) and 36 months (V08) were available. For the purpose of this study, which is to distinguish the SVA specific regulation from that associated with the *H1*/*H2* haplotype inversion, only individuals containing the *H1* haplotype (containing SVA_67) were considered for analysis.

### eQTL analysis

We used matrix eQTL to assess the genetic loci modulating expression of transcript variants^55^. For analysis, local (*cis*) and distant (*trans*) eQTL loci were documented, with a threshold on 1M bp for distant loci. An additive linear model with covariates, age and sex was applied, with FDR threshold 0.05. Additionally, effect-size estimates as beta values or slope coefficients, transcript and gene ID among others were reported. FDR was used for correction for multiple testing and only results passing this threshold are reported.

### PCR for SVA_67 VNTR genotyping

For genotyping of the SVA_67 VNTR, gDNA preparations from the PPMI cohort were used. For the purpose of this study, only individuals characterised by the SVA_67 associated *H1*/*H1* haplotype were selected for analysis. 91 individuals were genotyped by PCR. For amplification, nested PCR was applied. In the first reaction the following reagents (final concentration) were used: KOD Hot Start buffer (1x, Merck), MgSO_4_ (1.5 mM, Merck), dNTPs (0.2 mM each, Merck), Betaine (1 M, Sigma), Primer SVA_67_Fw (5’-AGTTGACCGTAATGTGAGCACT-3’) and SVA_67_Rv (5’-AGCCCCACAGGTAATACTTAATGA-3’) (0.3 µM, Sigma), KOD Hot Start DNA Polymerase (0.02 U/µl), gDNA template (0.25 ng/µl), made up with nuclease-free water to a final volume of 20 µl. Amplification reactions were performed using the SimpliAmp™ Thermal Cycler (Applied Biosystems) following the programme: 95 °C for 2 min, 95 °C for 20 s, 62 °C for 10 s and 70 °C for 50 s, repeating steps 2–4 34 more times. For the second reaction, 1µl of the PCR product from the first reaction was used using the primers SVA_67_int (5′-TCTACACAGACACGGCAACC-3′) and SVA_67_1 (5′-TCGAGACTAACCTGACCGGTG-3′), and same reagents and concentrations applied. The programme was as followed: 95 °C for 2 min, 95 °C for 20 s, 64 °C for 10 s and 70 °C for 10 s, repeating steps 2–4 34 more times. Products were visualised on 1–2% agarose gels (stained with ethidium bromide) using the BioDoc-It Imaging System (UVP).

### Generation of tagging SNPs for SVA_67 VNTR alleles and bioinformatic genotyping of SVA_67 alleles

For the generation of tagging SNPs for SVA_67 VNTR alleles 1, 3 and 4, SNPs for 1Mb surrounding SVA_67 and proxy SNPs for the SVA alleles were uploaded into haploview software (downloaded from the Broad Institute web page http://www.broadinstitute.org/scientific-community/science/programs/medical-and-population-genetics/haploview/downloads). Six SNPs (rs2532240, rs2732592, rs12942899, rs72836333, rs117464802 and rs118144601) were identified which define specific SVA_67 allele-containing haplotypes. Plink (v.1.07)^56^ was used to call haplotypes for 90 individuals of known SVA_67 alleles. The r^2^ threshold set to > 0.8 to identify SNPs in linkage disequilibrium with each other which were then correlated for the SNPs corresponding to the SVA_67 alleles to determine if the SVA is being tagged and inherited in a set haplotype.

### Cell culture

HEK293 cells (ATCC CRL-1573), HeLa (ATCC CCL-2) and SK-N-AS (ATCC CRL-2137), obtained from ATCC, were used for luciferase reporter assays. The cells were cultured in Dulbecco's Modified Eagle’s Media (DMEM) (Gibco) containing 4.5 g/L d-glucose and 200 mM l-glutamine (Gibco), supplemented with 10% foetal bovine serum (Gibco), penicillin/streptomycin (100 U/ml, 100 mg/ml) (Sigma) and 1% (v/v) 100 mM sodium pyruvate (Sigma). Culture medium for HeLa and SK-N-AS also contained 1% (v/v) MEM non-essential amino acids (Sigma, M7145). Cells were grown in a humidified incubator at 37 °C and 5% CO_2_.

### Generation of SVA_67 reporter gene constructs

Full-length SVA_67 sequences were amplified from genomic DNA by PCR using the primers SVA_67_1 (5′-TCGAGACTAACCTGACCGGTG-3′) and SVA_67_2 (5′-CAGGTTAGTCTCAAGATGTTC-3′) with reaction conditions as described before. Cycling conditions were as follows: 95 °C for 2 min, 95 °C for 20 s, 60 °C for 10 s and 70 °C for 10 s, repeating steps 2–4 34 more times. For luciferase assays, allele 1, 3 and 4 of SVA_67 were analysed. Corresponding PCR products of 701 bp (allele 1), 930 bp (allele 3) and 1059 bp (allele 4) were purified from the gel using the QIAquick Gel Extraction Kit (Qiagen) and initially subcloned into the intermediate pCR™-Blunt vector (Invitrogen) using the Zero Blunt™ PCR Cloning Kit (Invitrogen) following the manufacturer’s guidelines. The restriction enzymes *SpeI* and *XbaI* were used to digest the intermediate vectors and corresponding SVA_67 allele inserts were cloned upstream of the SV40 minimal promoter of the reporter gene vector pGL3-Promoter (pGL3-P) (Promega), previously digested with *NheI*. In addition, SVA_67 allele 3 was cloned into pGL3-Control (C) vector. For this, SVA-containing pGL3-P constructs and empty pGL3-C vector were digested with *SacI* and *XhoI* to clone full-length SVA-F (from *INPP5F* locus) and truncated SVA_67 allele 3 into pGL3-C. T4 DNA ligase (NEB) was used to complete the ligation process. All corresponding cloning steps were performed using Subcloning Efficiency™ DH5α Competent Cells (Invitrogen), grown in LB medium supplemented with the appropriate antibiotic (100 μg/mL ampicillin for pGL3-P/-C or 50 μg/mL kanamycin for pCR-Blunt). Final Plasmid DNA preparations used for transfection experiments were extracted using the Plasmid Maxi Kit (Qiagen) following the manufacturer’s protocol.

### Cell transfection and luciferase reporter gene assay

To characterise the properties of the different SVA_67 alleles to act as regulatory domain, 100,000 HEK293, HeLa and SK-N-AS cells, respectively were seeded per well in a 24-well-plate. Twenty-four hours later, cells were co-transfected with 1 µg of the corresponding reporter gene plasmid (encodes Firefly luciferase) and 20 ng pRL-TK control vector (encodes Renilla luciferase to enable normalisation) using TurboFect™ transfection reagent (Thermo Fisher) according to the manufacturer's protocol. As a negative control, the vector pGL3-Basic (pGL3-B) was used which contains no promoter to express Firefly luciferase. Forty-eight hours post-transfection, the Dual-Glo luciferase reporter assay system (Promega), according to the manufacturer's instructions, was used to assess luciferase activity of reporter constructs (Biological replicate *n* = 3, technical replicate per assay *n* = 3). The ratios of relative light units (RLU) for Firefly and Renilla luciferase were calculated for each pGL3-P/-C/-SVA_67 condition and averaged which were used to calculate fold changes compared to the control (pGL3-P empty vector).

### Analysis of differential gene expression

Previously determined SVA_67 allele genotypes were combined with transcriptomic data obtained from the PPMI cohort in order to assess the association of the SVA_67 allele genotype with differential gene expression. Quantification of transcriptomic data on a transcript- and gene-level was performed by using the Salmon tool (https://salmon.readthedocs.io). To import the Salmon-generated quant files into *R*, the *tximport* function from the *tximport* package^57^ was applied. Raw counts were extracted with the *DESeqDataSetFromTximport* function and normalised using the median-of-ratios method, included in the *DESeq2* package^58^. The *DESeq2* package in *R* was also used to assess differences in gene expression profiles associated with different allele genotypes of SVA_67. The *ggplot2* package in *R* was used to visualise the results.

### Association of SVA_67 with clinical features and progression markers of PD

For association analysis, a combination of a linear mixed-effect model and longitudinal data for PD clinical and progression marker was used. In addition to BL, datasets up to five visits were used for analysis by applying the following model using the *nlme* package in R: lmerTest::lmer(response ~ R_SVA_67_allele * EVENT_ID + (1|PATNO),na.action = na.omit,data = PD). Within this analysis, categorical values were transformed to numeric variables using the “as.numeric” function and 114 clinical features/variables from the PPMI cohort were analysed. We performed linear regression using the *lm* function in R to determine the association of age at onset and VNTR length.

### Statistical analysis

For eQTL analysis, an additive linear model with covariates, age and sex was applied. For multiple testing corrections, FDR was used. In luciferase reporter gene experiments, the mean ratios of each experiment were compared using ANOVA with Bonferroni correction for multiple comparisons to calculate statistical significance between pGL3-P and pGL3-C and SVA_67 allele-containing pGL3-P/C constructs, respectively. For differential gene expression analysis in association with SVA_67 VNTR alleles, a non-parametric multiple comparisons test, Kruskal–Wallis, was employed to assess significant difference between all SVA_67 allele genotypes and visits. For pairwise comparisons, the non-parametric Wilcoxon comparison test was applied to calculate *P* values for differences between SVA_67 alleles. Generated *p*-values were adjusted for multiple comparisons using FDR. For correlation of SVA_67 alleles with progression markers, *P* values from linear mixed-model were corrected by FDR. Pairwise comparisons between SVA_67 VNTR alleles and progression marker was performed and *P* values were adjusted with Tukey for family-wise error rate.

### Supplementary Information

Supplementary Information 1.

Supplementary Information 2.

Supplementary Information 3.

Supplementary Figure 4.

Supplementary Figures.

## Data Availability

The datasets used and/or analysed during the current study are publicly available from the Parkinson’s Progression Marker Initiative after submitting an online application form (https://www.ppmi-info.org/access-data-specimens/download-data). The study was approved by the Murdoch University Human Research Ethics Committee (ethics number: 2020/040).
