# Supplementary material for: Deciphering the role of a SINE-VNTR-Alu retrotransposon polymorphism as a biomarker of Parkinson’s disease progression
Source: Sci Rep. 2024 May 13;14:10932. doi: 10.1038/s41598-024-61753-5 (PMC11091082; doi:10.1038/s41598-024-61753-5)
Supplement: Supplementary file 4 — Supplementary Figure 4. [file 41598_2024_61753_MOESM4_ESM.docx]

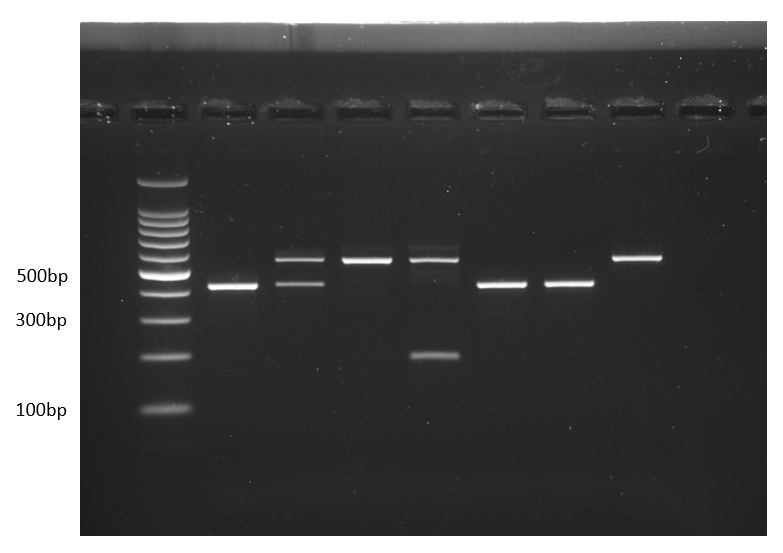


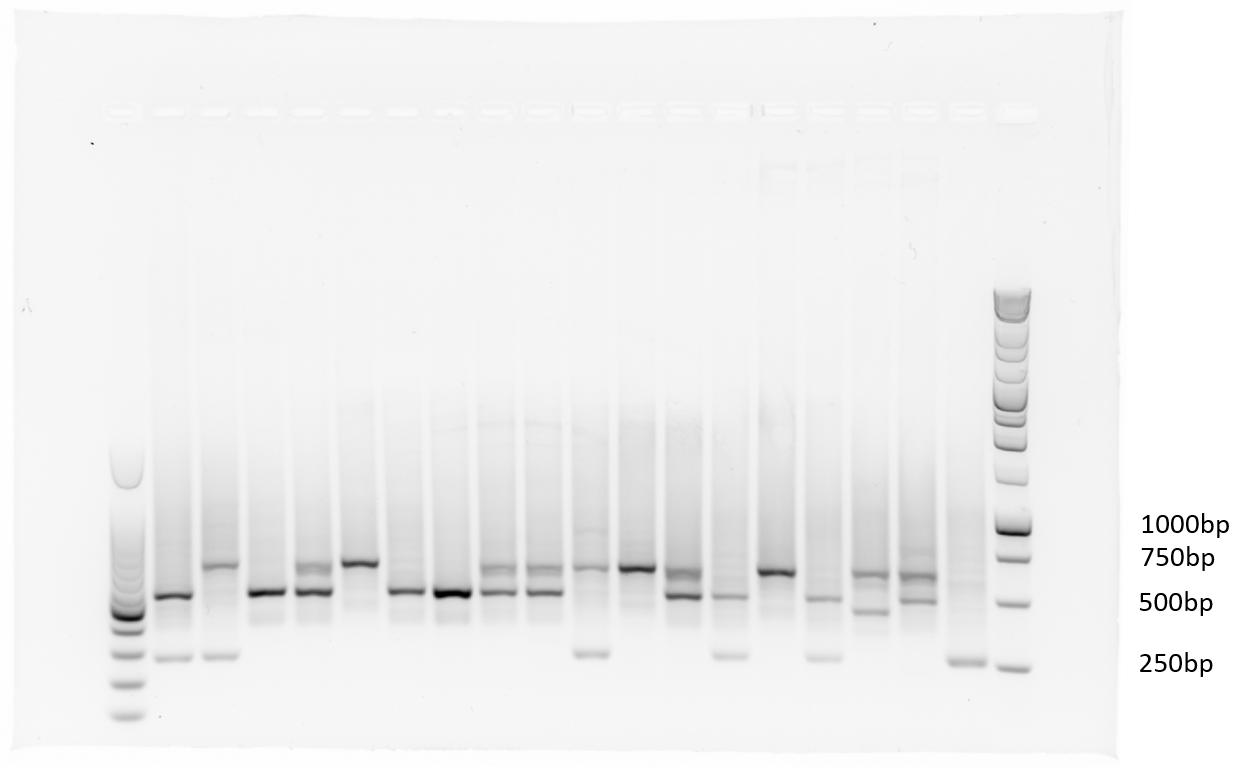


**Supplementary Figure 4:** Uncropped gels derived from PCR and gel electrophoresis of SVA_67 from a subset of the PPMI cohort. Cropped images are displayed in Fig. 3 and Supplementary Fig. 2, Additional File 2.
