# Supplementary material for: Deciphering the role of a SINE-VNTR-Alu retrotransposon polymorphism as a biomarker of Parkinson’s disease progression
Source: Sci Rep. 2024 May 13;14:10932. doi: 10.1038/s41598-024-61753-5 (PMC11091082; doi:10.1038/s41598-024-61753-5)
Supplement: Supplementary file 5 — Supplementary Figures. [file 41598_2024_61753_MOESM5_ESM.docx]

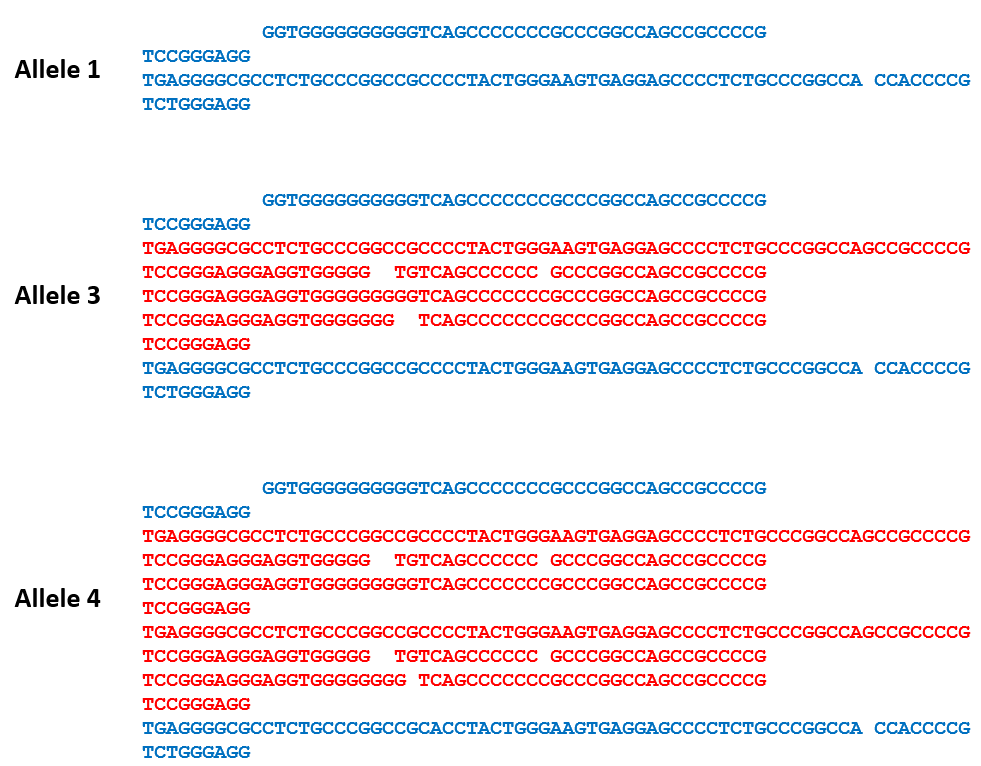


**Supplementary Figure 1: Alignment of SVA_67 VNTR allele sequences.** Four alleles of SVA_67 were identified by genotyping PCR. Excluding the SINE-R and poly-A tail, the different VNTR domains have a length of 128 bp (allele 1), 357 bp (allele 3) 485 bp (allele 4). Allele 2 was excluded from analysis.


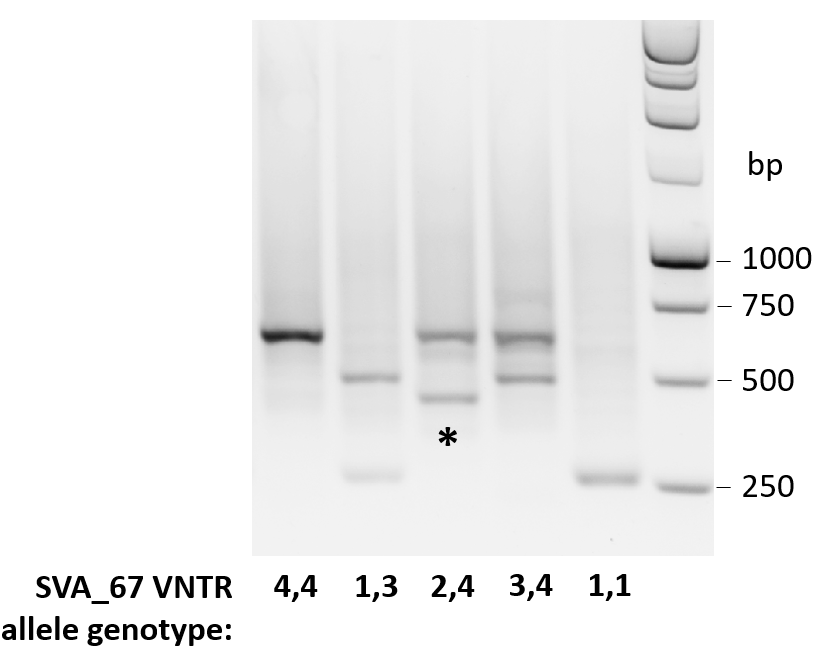


**Supplementary Figure 2:** PCR and gel electrophoresis of SVA_67 from a subset of the PPMI cohort. Representative gel image with expected amplicon size for SVA_67 alleles and corresponding SVA_67 genotypes are shown. Four SVA_67 alleles were identified (numbered 1–4 to reflect increasing length). Allele 2 is indicated by an asterisk.

**a**


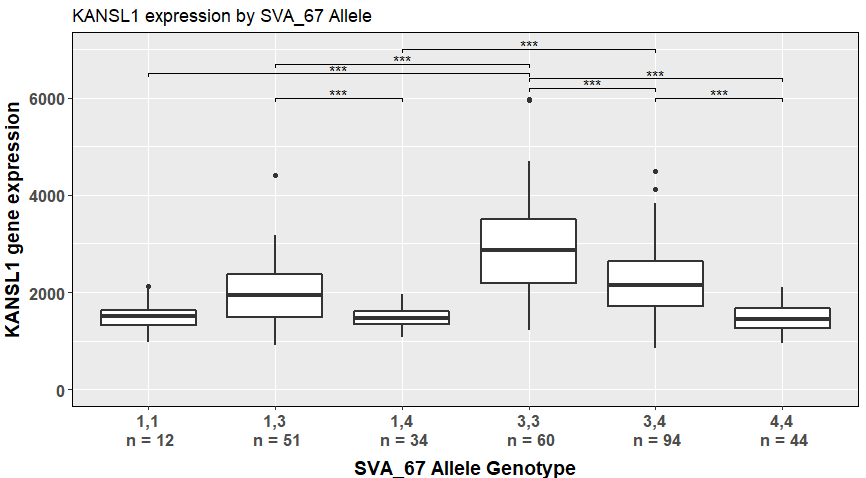


**b**


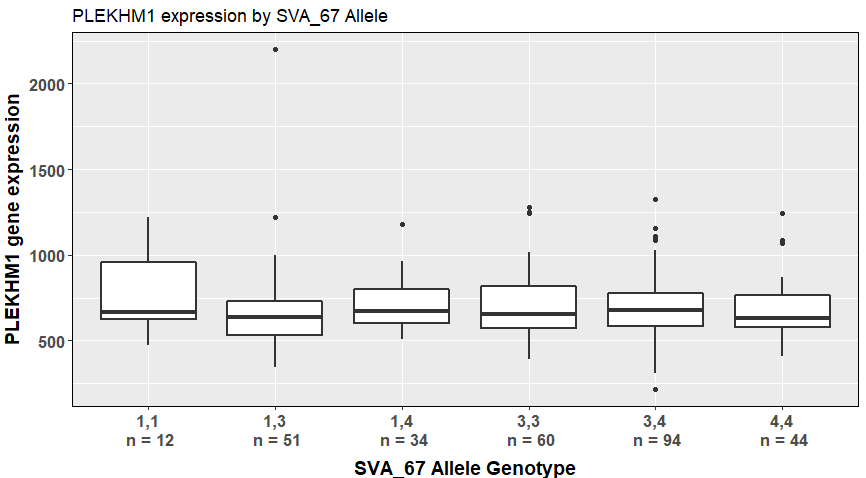


**c**


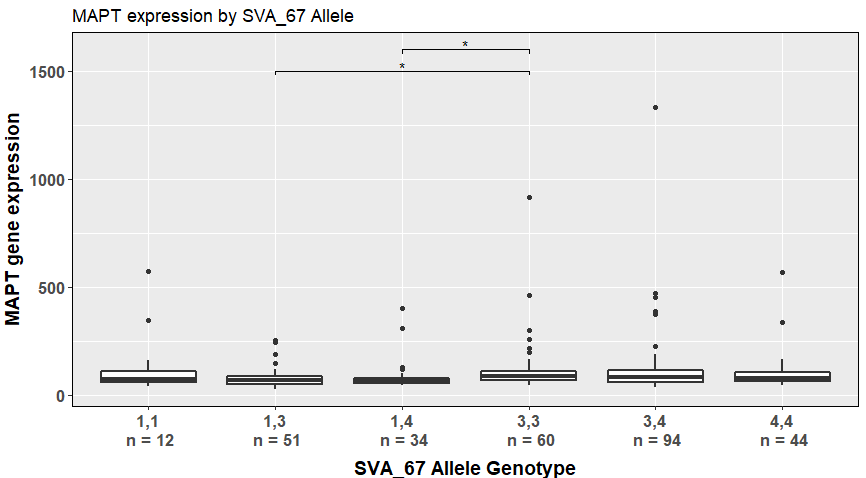


**Supplementary Figure 3**

**d**


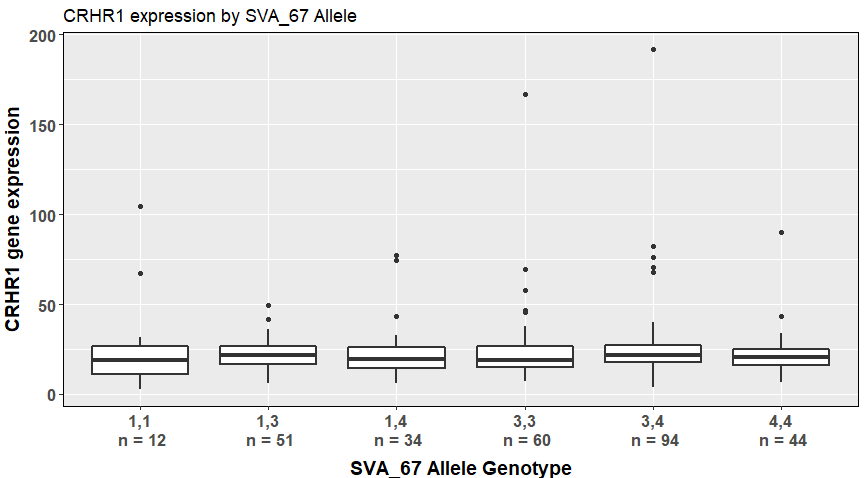


**e**


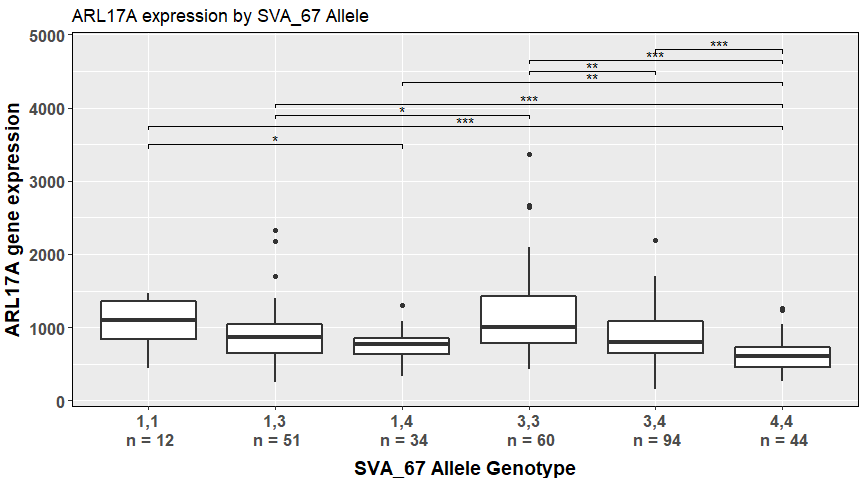


**f**


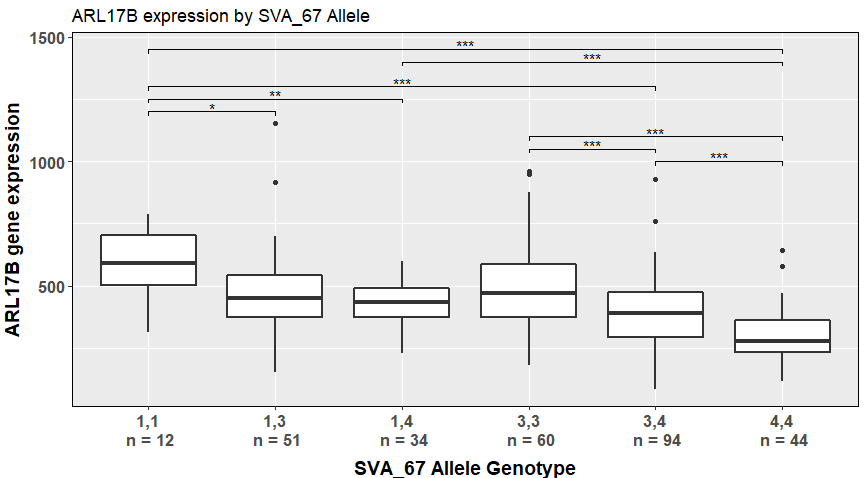


**Supplementary Figure 3**

**g**


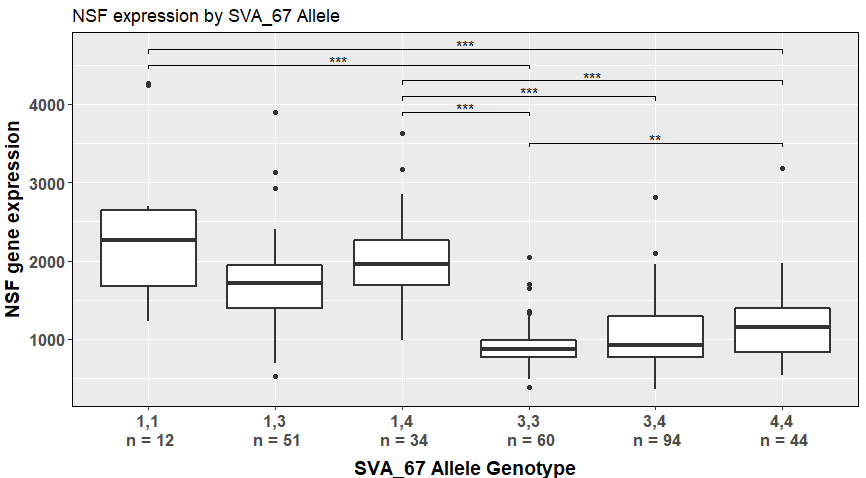


**Supplementary Figure 3: Association of SVA_67 allele genotype with expression of *KANSL1* (a), *PLEKHM1* (b), *MAPT* (c), *CRHR1* (d), *ARL17A* (e), *ARL17B* (f) and *NSF* (g) using datapoints from Baseline (0 months).** Statistically significant differences between SVA_67 allele genotypes were calculated using the non-parametric Kruskal-Wallis test. The Wilcoxon test was applied to calculate pairwise comparisons between group levels with corrections for multiple testing (FDR correction) indicated as asterisks. **P*<0.05, ***P*<0.01, ****P*<0.001.
